# Supplementary material for: The influence of college students’ aesthetic cognitions on aesthetic behaviours: The Chain mediation effect
Source: PLoS One. 2023 Dec 27;18(12):e0289666. doi: 10.1371/journal.pone.0289666 (PMC10752506; doi:10.1371/journal.pone.0289666)
Supplement: Attachment — Submitted filename: Response to Reviewers.docx [file pone.0289666.s003.docx]

Dear reviewer,

Thank you very much for your comments and professional advice. These opinions help to improve academic rigor of our article.Based on your suggestion and request, we have made corrected modifications on the revised manuscript, Meanwhile, the manuscript had be reviewed and edited by language servicices of AJE SEVIER. We hope that our work can be improved again. Furthermore, we would like to show the details as follows:

Reviewer #1: I thank the autors for addressing my previous commennts.

However, although the authors indicate that a section on limitations was added, I could not find (or see) this section!

A section on limitations should be inserted before the conclusions section.

Reviewer #2: The comments on methods section have been addressed and resolved. The comment on the literature review could be resolved by revising the way in which it was written, maybe to write it in a more critical way, by showing their voices too. However, it is only a recommendation because it has to do with the authors´writing style.
I recommend to accept the article.

The author’s answer: We have revised the sentence in the article. The details as follows:

**“6 Research limitations**
6.1 Research Sample
 In terms of sampling, limited by the researcher's time and ability, the sample of this study is only students of several universities in Shaanxi Province, China, and it is not possible to account for more Chinese college students in other provinces, which limits the interpretation of the results of the study and the inference.
6.2 Research Variables
 There are many factors that influence Chinese college students' aesthetic behavior, and this study is limited to two influences: aesthetic perception and aesthetic tendency. Other possible influencing factors, such as Chinese college students' personality traits, parents' income level, parents' attitudes toward art, the economic and cultural level of their cities, and the aesthetic environment, are not included in the variables of this study.
6.3 Research Method
 This study uses a questionnaire survey to collect information about personal background variables, aesthetic cognition, aesthetic tendency and aesthetic behavior of Chinese college students in Shaanxi Province; however, whether subjects can truly express and reflect the real situation may have a biased influence on the correctness of the results of the study.”

Thank you very much for you attention and time. Look forward to hearing from you.

Yours sincerely,

Qiao Qiao, Yongzhi Jiang

Krirk University

7 Oct., 2023
